# A New UPLC-MS/MS Method for the Characterization and Discrimination of Polysaccharides from Genus *Ephedra* Based on Enzymatic Digestions

**DOI:** 10.3390/molecules22111992

**Published:** 2017-11-17

**Authors:** Yong-Gang Xia, Tian-Long Wang, Li-Ming Sun, Jun Liang, Bing-You Yang, Hai-Xue Kuang

**Affiliations:** Key Laboratory of Chinese Materia Medica, Heilongjiang University of Chinese Medicine, Ministry of Education, Harbin 150040, China; yonggangxia@163.com (Y.-G.X.); wtl890322@163.com (T.-L.W.); llimingsun@163.com (L.-M.S.); lliangjunn@163.com (J.L.); ybywater@163.com (B.-Y.Y.)

**Keywords:** UPLC-MS/MS, polysaccharides, genus *Ephedra*, enzymatic digestion

## Abstract

*Ephedra sinica* polysaccharides have been reported to possess important activities, so quality evaluation of polysaccharides from the genus *Ephedra* is urgent. In this study, enzymatic digestions were performed to establish multiple saccharide fingerprints by ultra-performance liquid chromatography with electrospray ionization triple quadrupole linear ion trap mass spectrometry (UPLC-ESI-TQ-MS/MS) based on a multiple-reaction monitoring in negative mode. Under optimum UPLC-ESI^-^-TQ-MS/MS conditions, excellent separation and quantification of 21 constituents were achieved within 20 min on a solid core column with a 1.6 μm particle using pre-column derivatization with a PMP reagent. This method, coupled with enzymatic digestions and principal component analysis, has been successfully applied to characterize and discriminate *Ephedra* polysaccharides attributed to different species and plant parts. The results suggest that the proposed analytical strategy could achieve a quality evaluation of plant polysaccharides from traditional Chinese medicines.

## 1. Introduction

Mahuang is a famous traditional Chinese medicine (TCM) that has been used for thousands of years for the treatment of allergies, asthma, pneumonia, bronchitis, hay fever, and colds [1]. According to the 2015 edition of the Chinese pharmacopoeia, Mahuang is dry stems of *Ephedra sinica* Stapf, *E. intermedia* Schrenket C. A. Mey. and *E. equisetina* Bge., while Radix Ephedrae is recorded to be another different TCM, which is the dry roots or rhizoma of *E. sinica* and *E. intermedia* in the official pharmacopoeia of China [2]. Recently, some reports have demonstrated that the stems of *E. sinica* contain a high amount of polysaccharides, ranging from 3% to 5% of the total weight [3,4]. In addition, *E. sinica* stem polysaccharides showed obviously immunosuppressive effects by a carbon clearance test, delayed type hypersensitivity reaction, and humoral immune response in vivo [4,5,6]. However, so far there is no published information on discrimination of *Ephedra* polysaccharides from different species and plant parts. Therefore, it is essential to develop an effective method for quality evaluation of *Ephedra* polysaccharides.

A variety of chromatographic techniques such as gas chromatography (GC) [7], capillary electrophoresis (CE) [8], high-performance liquid chromatography (HPLC) [9,10], and high-performance anion exchange chromatography (HPAEC) [11,12] were previously considered as useful tools for the determination of carbohydrates. Although existing techniques are increasingly mature and gradually improving, they still face many problems and challenges. The limit of quantification of the GC, HPLC-UV, and CE-UV method is too restrictive and cannot be used in trace quantification of carbohydrates. HPAEC can achieve more satisfactory separation than HPLC techniques, but the epimerization and degradation can be issues at high pH [11,12]. Except for HPAEC, most of the LC and CE techniques often use labeling with either fluorescence or UV tags for enhanced detection. The reagent 1-phenyl-3-methyl-5-pyrzolone (PMP) has been widely used for composition analysis of plant polysaccharides [3].

Carbohydrates encompass a number of homologues with similar structures, so the analysis of carbohydrates inevitably requires high-resolution separation techniques. With the progress of chromatographic techniques, ultra-performance liquid chromatography (UPLC) was developed with a sub-2 μm diameter, which shortened the analysis time and provided high resolution and sensitivity separation, especially tandem mass spectrometry (MS/MS). Although UPLC-MS/MS have been applied to analyze plant polysaccharides, most reports are limited to several monosaccharides [13,14].

In this study, UPLC with triple quadrupole linear ion trap mass spectrometry (TQ-MS/MS) was firstly developed for simultaneous analysis of 21 carbohydrates and one degradation product (5-HMF, 5-hydroxymethyl furfural) based on a pre-column PMP derivatization. Elution of all eight neutral sugars, two uronic acids, three amino sugars, and two acetyl amino sugars, was then used to evaluate the performance of this method. Finally, UPLC-ESI^−^-TQ-MS/MS coupled with enzymatic digestions and principal component analysis (PCA) has been successfully applied to analyze and characterize 20 *Ephedra* polysaccharide samples attributed to different species and plant parts. The results showed that the proposed analytical methods provided reasonable insight and guidance for quality evaluation of plant polysaccharides from the genus *Ephedra*.

## 2. Experimental

### 2.1. Materials and Reagents

*E. sinica*, *E. intermedia*, and *E. equisetina* were purchased in September 2012 from Datong, Shanxi Province, China and identified by Professor Zhen-Yue Wang of Heilongjiang University of Chinese Medicine. The voucher specimens (20120905) were deposited at the herbarium of Heilongjiang University of Chinese Medicine, Harbin, China.

d-galacturonic acid (GalUA), d-glucuronic acid (GlcUA), d-fucose (Fuc), l-rhamnose (Rha), d-glucose (Glc), d-galactose (Gal), d-mannose (Man), l-arabinose (Ara),d-ribose (Rib), *N*-Acetyl-d-(+)-glucosamine (GlcNAc), *N*-Acetyl-d-(+)-galactosam-ine (GalNAc), d-glucosamine hydrochloride (GlcN), d-galactosamine hydrochloride (GalN), d-mannosamine hydrochloride (ManN), 5-hydroxymethyl furfural (5-HMF), lactose, maltotriose, maltotetraose, maltopentaose, maltohexaose, and maltoheptaose were purchased from Sigma- Aldrich (St. Louis, MO, USA). D_2_-Glc, 1-phenyl-3-methyl-5-pyrazolone (PMP), ammonium formate (AF), and formic acid (FA) were also obtained from Sigma-Aldrich. Pectinase, α-amylase, and cellulose were purchased from Sigma-Aldrich; xylanase and *endo*-1,3-β-glucanase were obtained from Megazyme. Water was obtained from a Milli-Q purification system (Millipore, Bedford, MA, USA). All other chemicals were of the highest analytical grade.

### 2.2. Preparation of Polysaccharides from Genus Ephedra

The dried sample materials (100 g) were extracted three times with 10-fold volume of distilled water under reflux for 3 h each time. The filtrate of the obtained extract was condensed under a vacuum to syrup and precipitated with 95% ethanol added to a final concentration of 80%. After standing for 24 h at 4 °C, the precipitate was collected and washed with anhydrous ethanol, then dried. The residue was re-dissolved in water, and then, after centrifugation (4000 rpm for 15 min), the supernatant was dialyzed (cut-off *Mw* 3500 Da) against tap water for 48 h and distilled water for 48 h and lyophilized to yield crude polysaccharides. The crude polysaccharides, which were prepared in duplicate, were obtained for further analysis.

### 2.3. Specific Enzymatic Digestion of Ephedra Polysaccharides

Polysaccharide solutions (5 mg/mL, 200 μL) were mixed with specific enzymes (the final concentrations of pectinase, *endo*-1,4-β-xylanase, cellulose, β-(1→3)-d-glucanase and *α*-amylase were 30 U/mL, 10 U/mL, 0.3 U/mL, 0.2 U/mL and 30 U/mL, respectively) in a 1.5-mL centrifuge tube and digested overnight (≥15 h) at 50 °C. Then the mixtures were heated at 80 °C for 20 min to stop the enzymatic digestion. After centrifugation (12,000 rpm/min) at room temperature for 20 min, the supernatants were evaporated to dryness with a nitrogen evaporator at 70 °C and then used for derivatization with PMP. Moreover, the blank control was treated as described above without enzymes.

### 2.4. Derivatization with PMP Reagent

PMP derivatization of carbohydrates was carried out as described previously with proper modifications [3]. Briefly, PMP was prepared in methanol at a final concentration of 0.5 M. One hundred microliters of each individual standard monosaccharide and oligosaccharide, mix standard solutions, and the hydrolyzed polysaccharide samples were placed in 1.5-mL centrifuge tubes. Then, 0.5 M PMP (100 μL) and ammonia (200 μL) were added. D_2_-Glc as an internal standard (2 M) was added to each sample (2 μL). Each mixture was allowed to react for 20 min in a 70 °C water bath, then cooled to room temperature and neutralized with 200 μL of formic acid. The solution was directly filtered through a 0.22-μm membrane and diluted with deionized water before UPLC-TQ-MS/MS analysis.

### 2.5. UPLC Apparatus and Conditions

An ACQUITY ultra-performance liquid chromatographic system (Waters, Milford, MA, USA) was applied. The chromatographic separation was conducted on a cortecs UPLC C_18_ column (1.6 μm, 2.1 × 100 mm) equipped with a cortecs UPLC C_18_ guard column (1.6 μm, 2.1 × 5 mm). The binary mobile phase was composed of acetonitrile as solvent A and deionized water with 10 mM AF as solvent B. Optimal run time for separation was 20 min using the following gradient program at a flow rate of 0.3 mL/min and a column oven temperature of 30 °C: isocratic 13% A at 0–0.5 min; 0.5–2 min, linear from 13% to 16% A (*v*/*v*); 2–15 min, linear from 16% to 21% A (*v*/*v*); 15–16 min, linear from 21% to 60% A (*v*/*v*); 16–17 min, linear from 60% to 90% A (*v*/*v*); followed by column re-equilibration using 13% A at 17–20 min. The injection volume was set to 2 μL.

### 2.6. MS Apparatus and Conditions

The MS detection was performed using an API 4000 Qtrap (AB Sciex, Foster City, CA, USA) equipped with an electrospray ionization (ESI) interface, and operated in negative ion mode with multiple reaction monitoring (MRM). Turbo V ion source parameters were common to all analytes, as follows: the capillary voltage was −4.5 kV, and the source temperature was at 550 °C. The curtain gas and collision gas setting were 30 psi and 10 psi, respectively. The pressure for nebulization gas and vaporization gas setting were 55 psi. The declustering potential (DP) and collision energy (CE) were optimized for each analyte.

### 2.7. Data Processing

Evaluation of the different polysaccharides from genus *Ephedra* could be facilitated using multivariate statistical analysis. PCA was applied to visualize the data structure, classify the samples according to their components and detect corresponding markers. The PCA was carried out using SPSS version 16.0 (SPSS, Inc., Chicago, IL, USA).

## 3. Results and Discussion

### 3.1. Derivatization and MS Conditions

**Figure 1:**
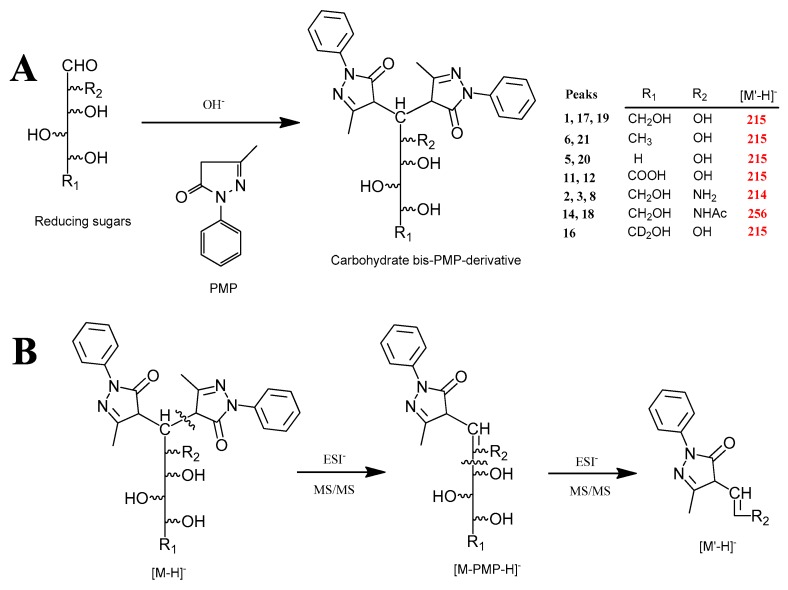
(**A**) Condensation reaction with PMP; (**B**) characteristic fragmentation pathways of bis-PMP derivatives of monosaccharides in negative ESI-MS/MS mode. Peaks were as follows: **1**, Man; **2**, GlcN; **3**, ManN; **5**, Rib; **6**, Rha; **8**, GalN; **11**, GlcUA; **12**, GalUA; **14**, GlcNAc; **16**, D_2_-Glc; **17**, Glc; **18**, GalNAc; **19**, Gal; **20**, Ara; **21**, Fuc.

In order to achieve a fast and accurate sample preparation, a simplified PMP-derivatization procedure was developed. The PMP reaction mechanism was illustrated in Figure 1A. This modified procedure was characterized by extreme simplification without using any liquid–liquid extraction and concentrated steps by comparison with our previous reports [3]. The resulting solution was directly filtered and diluted with deionized water before UPLC-ESI^−^-TQ-MS/MS. Furthermore, the comparative experiments between before and after liquid–liquid extraction showed that the simplified workflow did not affect the chromatographic separation and MS signal intensities at all. This simplified sample preparation was applied successfully in the current study.

Regarding MS detection, carbohydrate PMP derivatives could be monitored under both ESI^−^ and ESI^+^ modes. In the present work, much less noise was observed in the ESI^−^ than ESI^+^ modes. Furthermore, the [M − H]^−^ precursor ions were more intense for all analytes than [M + H]^+^ and [M + Na]^+^, so [M − H]^−^ precursor ions were used for the detection of the carbohydrate PMP derivatives. Taking collision-induced dissociations of deprotonated PMP-labeled monosaccharides as examples, peaks **1**–**3**, **5**, **6**, **8**, **11**, **12**, **14**, **16** and **17**–**21** produced similar fragmentation patterns. The typical ESI^−^ fragmentation pathway is illustrated in Figure 1B. The [M − H]^−^ precursor first experienced the loss of a PMP unit to afford a [M − PMP − H]^−^ ion, which further produced *α*-cleavage to give characteristic [M′ − H]^−^ ions at *m*/*z* 214, 215 or 256. This [M′ − H]^−^ was considered to be a characteristic product ion based on the fact that this fragment was always stable with prominent intensity when using a specific collision energy. Therefore, the experimental MRM transition used those intact deprotonated *m*/*z* values of precursors (Q_1_) and characteristic fragments [M′ − H]^−^ as product ions (Q_3_) for accurate detection of bis-PMP derivatives of monosaccharides—**3**, **5**, **6**, **8**, **11**, **12**, **14** and **16**–**21**. As a contrast, bis-PMP derivatives of oligosaccharides **4**, **7**, **9**, **10**, **13** and **15** showed more complex fragmentation mechanism in ESI^−^-MS/MS mode, so the most intense product ion was chosen as Q_3_.

Finally, the optimal DP and CE values for all MRM transitions (**1**–**22**) are summarized in Table 1. Another advantage of the current method in comparison with traditional HPLC-UV methods is that it could effectively eliminate the interference of the PMP ion peak using UPLC-ESI^−^-TQ-MS/MS with MRM detection.

### 3.2. Quantification and Validation

The chromatographic conditions were optimized to achieve the best resolution of various saccharides of interest within a short analysis. Some carbohydrate PMP derivatives might have different structures, but have the same precursor ion and daughter ion. So it is difficult to fully separate them by common HPLC techniques. Thus, UPLC-ESI^−^-TQ-MS/MS was explored to separate and detect carbohydrates. To establish an ideal separation, the chromatographic parameters involving column types, additives of mobile phases, flow rates and column temperatures were optimized.

#### 3.2.1. Effects of Columns

The UPLC method was developed to achieve a possible separation of various carbohydrate PMP derivatives of interest. The result showed that some tested PMP derivatives were inadequately separated (**2** and **3**, **5** and **6**) by BEH C_18_ (2.1 × 100 mm, 1.7 μm) or available at a lower MS response intensity (**1**, **2**, **3**, **8**, **11**, **12** and **16**–**19**) using the HSS T3 column (2.1 mm × 100 mm, 1.8 μm) and ACE C_18_ (150 × 2.1mm, 3 μm) or a longer retention time up to 20–30 min (**16**–**19**) employing a HSS T3 column (2.1 mm × 150 mm, 1.8 μm) and BEH C_18_ (2.1 × 100 mm, 1.7 μm). Therefore, a cortecs UPLC C18 (2.1 × 100 mm, 1.6 μm) column was used in this work to detect PMP derivatives **1**–**22**. It was found that this specific column could provide high column efficiency and good MS response while retaining a relatively short run time.

#### 3.2.2. Effects of Additives

To obtain chromatograms with good resolution and high intensity, various additives such as acetic acid, FA, and AF were investigated in the pre-experiment, and AF was shown to give the best separation and peak shape. The effects of additive AF concentrations (8 mM, 10 mM, 15 mM and 10 mM AF with 0.1% FA) were also investigated on resolutions and MS response intensities. The results showed the moderate 10 mM AF showed the highest MS intensity for most of the peaks tested. If 10 mM AF with a lower pH was applied, it seemed to make some peaks co-eluted, such as 1–3 and 5–8. Therefore, 10 mM AF was used for further, optimized steps.

#### 3.2.3. Effects of Column Temperature and Flow Rate

The effects of different column temperatures (25 °C, 30 °C and 35 °C) were assayed. Generally, all carbohydrate PMP derivatives tested were eluted to be a sharp peak at column temperatures of 25–35 °C; there was no obvious effect on the peak symmetry and resolution of eluted analytes. However, peak responses were much better when the column temperature was applied at 30 °C. Therefore, the column temperature of 30 °C was used for further optimization steps. The effect of different flow rates (0.2, 0.3 and 0.4 mL/min) has also been evaluated. After comprehensive consideration of peak resolutions, symmetries, retention time, and column pressures, 0.3 mL/min was selected as the optimum flow rate.

**Figure 2:**
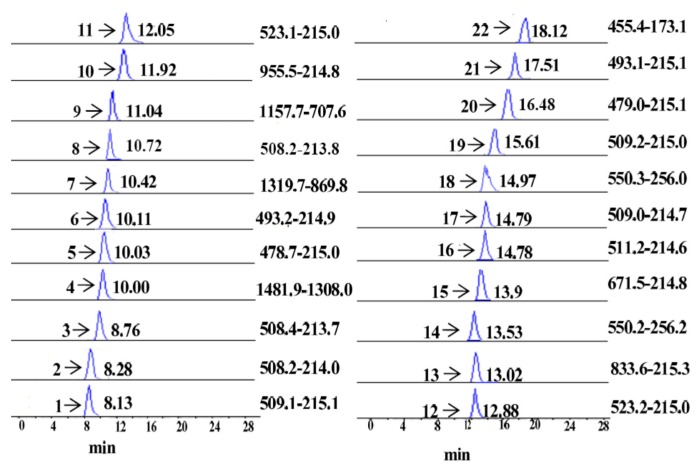
MRM ion chromatograms and 22 reference standards. Peaks **1**–**3**, **5**, **6**, **8**, **11**, **12**, **15** and **16**–**21** are the same as in Figure 1. Other peaks are as follows: **4**, maltoheptaose; **7**, maltohexaose; **9**, maltopentaose; **10**, maltotetraose; **13**, maltotriose; **15**, lactose; **22**, 5-HMF.

Under the above optimum conditions, the MRM chromatograms of reference standards **1**–**22** are given in Figure 2, which shows the complete baseline separation of all corresponding isomeric carbohydrate PMP derivatives, including **1**, **17** and **19** (Q_1_/Q_3_, 509/215), **2**, **3**, and **18** (Q_1_/Q_3_, 508/214), **5** and **20** (Q_1_/Q_3_, 479/215), **6** and **21** (Q_1_/Q_3_, 493/215), and **11** and **12** (Q_1_/Q_3_, 523/215). These targeted analytes include 14 monosaccharides (**1**–**3**, **5**, **6**, **8**, **11**, **12**, **14**, **17**–**21**), one internal standard compound (**16**), six oligosaccharides (**4**, **7**, **9**, **10**, **13** and **15**) and one degraded product (**22**). Although some peaks such as **1**, **2**, **5**, **6**, **10**–**14** and **16**–**18** were not fully separated in total ion chromatography (Supplementary materials Figure S1), they were still accurately quantified due to the presence of different MRM selections. It is worth mentioning that this is the first example on simultaneous analysis of eight neutral sugars (**1**, **5**, **6**, **16**, **17**, **19**–**21**), two uronic acids (**11** and **12**), three amino sugars (**2**, **3** and **8**), two acetyl amino sugars (**14** and **17**), six oligosaccharides (**4**, **7**, **9**, **10**, **13** and **15**), and one degradation product (**22**) using UPLC-ESI^−^-TQ-MS/MS.

### 3.3. Method Validations

The analytical procedure has been validated in order to confirm its reliability. Table 2 summarizes the calibration curves, LOD and LOQ values of the carbohydrate PMP derivatives analyzed by UPLC-ESI^−^- TQ-MS/MS with MRM mode. Since it is difficult to be precise about sample preparations due to the presence of derivatization procedures, the internal standard method is used. Here synthetic D_2_-Glc was chosen to be the internal standard in terms of different Q_1_/Q_3_ MRM selection. All the peaks showed good linearity (*R*^2^ > 0.99) in quite a wide concentration range. The LOD and LOQ were determined at a signal-to-noise (S/N) of about 3 and 10, respectively. The results showed that the LODs of monosaccharides and oligosaccharides were in the range of 3–48 nM and 12–780 nM, respectively, indicating that the sensitivity of the method was satisfactory. The intra-day and inter-day RSD values of the analytes were all less than 4.86%, implying good precision and reproducibility. For stability testing, the same sample solution was analyzed with 21 analytes every 4 h over 24 h with RSD less than 4.03%, which indicated that the sample was stable over two days under experimental conditions.

Furthermore, recovery experiments were performed in order to investigate the accuracy of the method. Known amounts of carbohydrate solutes were added to the sample with same procedures including extraction and hydrolysis, and the resulting spiked sample was subjected to the entire analytical sequence. The recovery rate of the method was 94.37%–104.37%, with RSD less than 4.23%, suggesting that the method is accurate and practical for the composition analysis in plant polysaccharides.

The occurrence of matrix effects in UPLC-ESI^−^-TQ-MS/MS is well known and has an important impact on the determination of the constituents in complex plant samples. A matrix effect was obtained by relative recoveries: relative recoveries = (sample contents after adding − original contents)/contents of standard solutions for adding. The relative recoveries for all compounds ranged between 94.94% and 100.75%, thus showing the minimal matrix suppression or enhancement of this method.

### 3.4. Application to Real Samples

**Figure 3:**
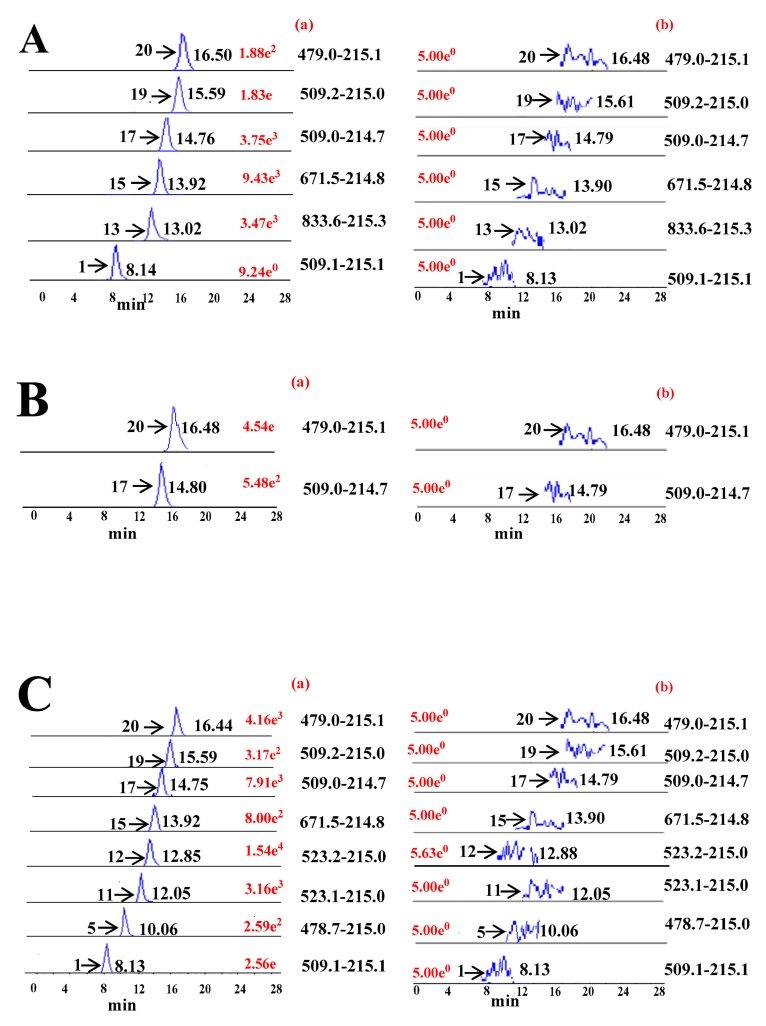

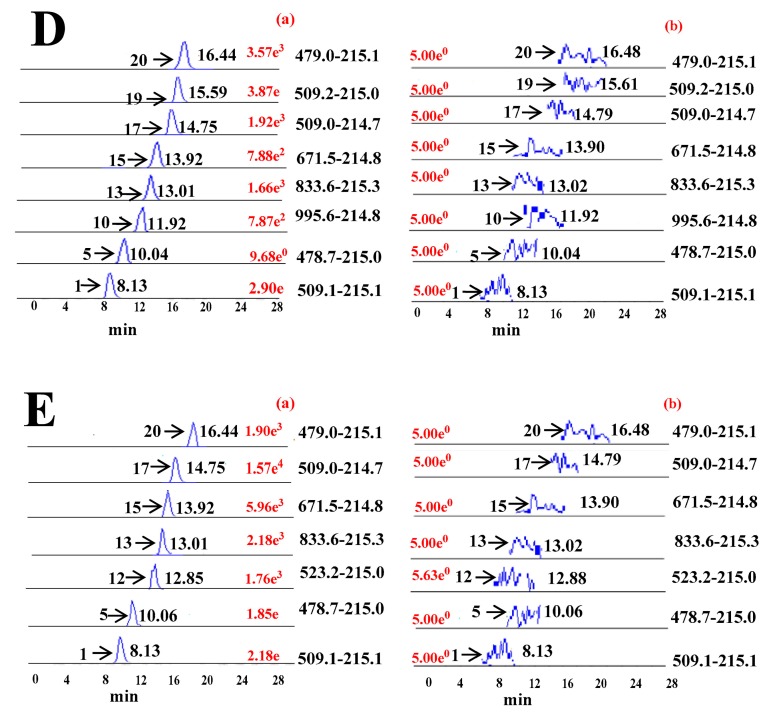
Typical MRM ion chromatograms of *E. sinica* root polysaccharides treated by multiple enzymatic digestions. (**A**), α-amylase; (**B**), β-(1→3)-d-glucanase, (**C**), pectinase; (**D**), cellulose; (**E**), endo-1,4-β-xylanase. (**a**,**b**) were due to polysaccharide samples with and without enzymatic digestions, respectively.

After PMP derivatization of *Ephedra* polysaccharide-treated enzymatic digestions, it was directly injected and separated under the optimum conditions described above. The results of multiple enzymatic digestions are shown in Supplementary materials Table S1. Figure 3A–E shows typical MRM ion chromatograms of *E. sinica* root polysaccharides using UPLC-ESI^−^-TQ-MS/MS before and after multiple enzymatic digestions, respectively. When the *α*-amylase was used for the treatment of polysaccharides from genus *Ephedrae*, Ara (**20**), Glc (**17**), Gal (**19**), Man (**1**), lactose (**15**), and maltotriose (**13**) were readily liberated (Figure 3A). The β-(1→3)-d-glucanase digestion could produce both Ara (**20**) and Glc (**17**) (Figure 3B), while the pectinase digestion enabled Ara (**20**), Glc (**17**), GlcUA (**11**), GalUA (**12**), and lactose (**15**) to be liberated from the core skeletons of *Ephedrae* polysaccharides (Figure 3C). Meanwhile, a large amount of Glc (**17**), Ara (**20**), lactose (**15**), and maltotriose (**13**) were freed using the cellulose digestion of *Ephedra* polysaccharides (Figure 3D). If the endo-1,4-*β*-xylanase was applied, the predominant compositional saccharides were detected to be Ara (**20**), Glc (**17**), lactose (**15**), maltotriose (**13**), and maltotetraose (**10**) (Figure 3E). Note also that a tetrasaccharide (Glc^1^(α)→^4^Glc(α)→^4^Glc(α)→^4^Glc(α)) residue should be linked in the side chain. The above evidence showed that *Ephedra* polysaccharides from different species and parts (stems or roots) may possess similar structural characteristics. Therefore, it is difficult to directly classify and discriminate these polysaccharides from genus *Ephedra* by eye.

### 3.5. Discrimination of Ephedra Polysaccharides Based on PCA

**Figure 4:**
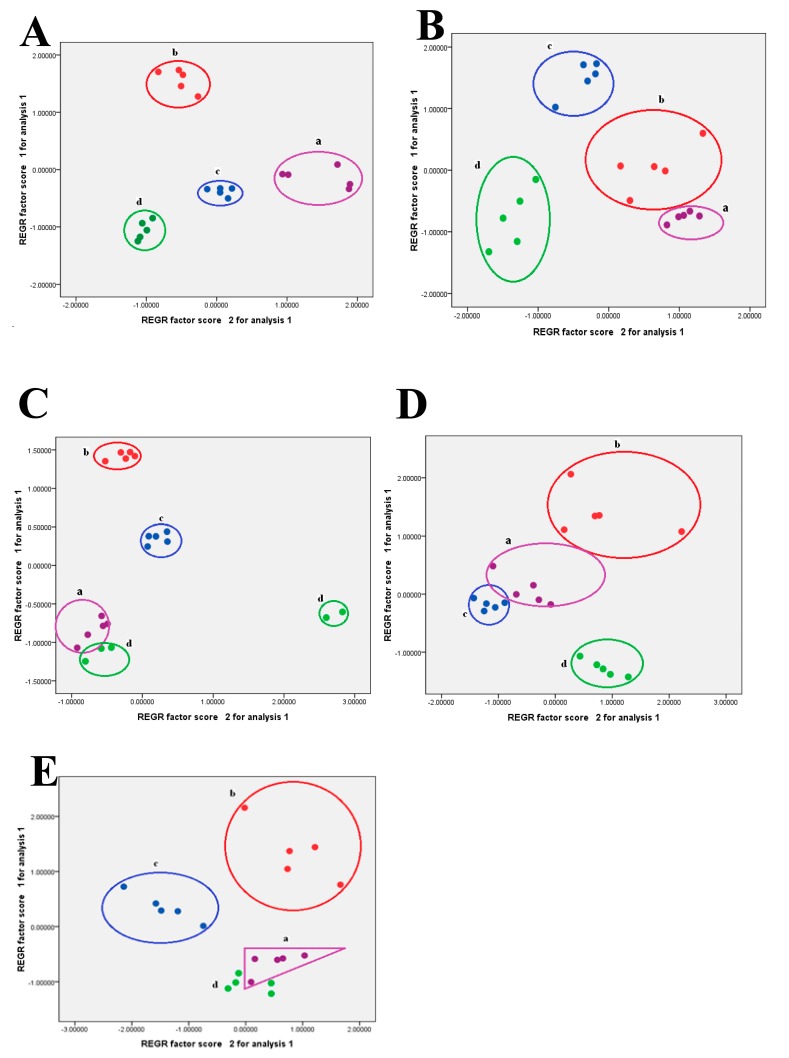
PCA score plots of 20 polysaccharide samples from multiple enzymatic digestions. (**A**) α-amylase; (**B**) β-(1→3)-d-glucanase, (**C**) pectinase; (**D**) cellulose; (**E**) *endo*-1,4-β-xylanase. (**a**) *E. sinica* (stem); (**b**) *E. sinica* (root); (**c**) *E. intermedia* (stem); (**d**) *E. equisetina* (stem).

As shown in Figure 4A,B,D, 20 *Ephedra* polysaccharide samples from different species and plant parts were well distinguished using *α*-amylase, β-(1→3)-d-glucanase, and cellulose enzymatic digestions, respectively. It was noticeable that samples 1–20 were clustered into four groups, **a**–**d**, which were unambiguously identified as polysaccharides from *E. sinica* stem, *E. sinica* root, *E. intermedia* stem, and *E. equisetina* stem, respectively. The principal components of enzymatic digestions (endo-1,4-β-xylanase and pectinase) had a minor effect on the model. The typical PCA scores from endo-1,4-β-xylanase and pectinase digestion are illustrated in Figure 4C,E, respectively.

The PCA loadings (not shown) were used to identify the differential components accountable for the separation among different groups. Ara, Gal, Glc, lactose, Man, and maltotriose were the principal components in classifying these samples for the α-amylase digestion of *Ephedra* polysaccharides. The Ara and Glc may play essential roles in the discrimination of *Ephedra* polysaccharides treated by the β-(1→3)-d-glucanase digestion. Meanwhile, the lactose, maltotriose, Ara, GalUA and Glc could be considered marker components to differentiate these samples using the cellulose digestion. These results further indicated that *Ephedra* polysaccharides, which were digested by *α*-amylase, β-(1→3)-d-glucanase, and cellulose, produced characteristic saccharide fingerprints. Therefore, UPLC-ESI^−^-TQ-MS/MS coupled with PCA could be used for differentiating *Ephedra* polysaccharides attributed to different species and plant parts through specific enzymatic digestions.

## 4. Conclusions

In this work, a reliable, simple, and sensitive UPLC-ESI^−^-TQ-MS/MS method was developed for the simultaneous analysis of 21 PMP derivatives characterized by the presence of seven neutral sugars, two uronic acids, three amino sugars, two acetyl amino sugars, six oligosaccharides, and one degradation product based on a solid core cortecs C_18_ column within 20 min. The proposed method minimizes sample handling, permits high-throughput analysis, and has been successfully applied to analyze 20 *Ephedra* polysaccharide samples from different species and plant parts. Multivariate statistical analysis results indicated that the specific enzymatic digestions (α-amylase, β-(1→3)-d-glucanase, and cellulose) could be used for distinguishing these polysaccharides from genus *Ephedra*. UPLC-ESI^−^-TQ-MS/MS, coupled with enzymatic digestions and multivariate statistical analysis, may be a powerful and useful approach for quality evaluation of plant polysaccharides from Traditional Chinese Medicine.

## Figures and Tables

**Table 1:** Mass spectrometry parameters used for analysis of targeted saccharides and internal standards (IS).

Peaks	Original *M_w_*	PMP Derivatives (*m*/*z*)	Q_1_ (*m*/*z*)	Q_3_ (*m*/*z*)	DP (V)	CE (V)
**1**	180.2	[M − H]^−^	509.1	215.1	−49.2	−28.1
**2**	215.6	[M − H]^−^	508.2	214.0	−65.9	−29.8
**3**	215.6	[M − H]^−^	508.4	213.7	−64.5	−26.0
**4**	1153.0	[M − H]^−^	1481.9	1308.0	−96.4	−35.1
**5**	150.1	[M − H]^−^	478.7	215.0	−58.4	−29.9
**6**	164.2	[M − H]^−^	493.2	214.9	−60.6	−29.0
**7**	990.9	[M − H]^−^	1319.7	869.8	−99.5	−61.2
**8**	215.6	[M − H]^−^	508.2	213.8	−67.9	−33.2
**9**	828.7	[M − H]^−^	1157.7	707.6	−90.3	−51.7
**10**	666.6	[M − H]^−^	995.6	214.8	−80.8	−52.6
**11**	212.2	[M − H]^−^	523.2	215.0	−55.9	−30.3
**12**	194.1	[M − H]^−^	523.1	215.0	−57.5	−47.4
**13**	504.4	[M − H]^−^	833.6	215.3	−98.4	−46.8
**14**	221.2	[M − H]^−^	550.2	256.2	−63.9	−32.9
**15**	360.3	[M − H]^−^	671.5	214.8	−61.2	−34.1
**16**	182.2	[M − H]^−^	511.2	214.6	−63.7	−28.0
**17**	180.2	[M − H]^−^	509.0	214.7	−59.9	−26.1
**18**	221.2	[M − H]^−^	550.3	256.0	−69.4	−34.1
**19**	180.2	[M − H]^−^	509.2	215.0	−65.0	−29.9
**20**	150.1	[M − H]^−^	479.0	215.1	−48.4	−33.9
**20**	150.1	[M − H]^−^	479.1	215.0	−63.1	−28.1
**21**	164.2	[M − H]^−^	493.1	215.1	−54.5	−30.4
**22**	126.1	[M − H]^−^	455.4	173.1	−60.9	−14.1

Peaks **1**–**3**, **5**, **6**, **8**, **11**, **12**, **15** and **16**–**21** were the same as in Figure 1. Other peaks are as follows: **4**, maltoheptaose; **7**, maltohexaose; **9**, maltopentaose; **10**, maltotetraose; **13**, maltotriose; **15**, lactose; **22**, 5-HMF.

**Table 2:** Summarization of calibration results, LOD, and LOQ values.

No.	Calibration Curves	*R*^2^	Linear Range (μM)	LOD (nM)	LOQ (nM)
**1**	*y* = 0.2634*x* + 0.1916	0.9938	0.02–49.60	3.00	8.00
**2**	*y* = 0.1721*x* + 0.0360	0.9982	0.19–49.60	24.00	58.00
**3**	*y* = 0.2135*x* + 0.2631	0.9931	0.05–49.60	12.00	34.00
**4**	*y* = 0.0002*x* + 0.0002	0.9995	3.10–49.60	780.00	2240.00
**5**	*y* = 0.4846*x* + 0.2466	0.9960	0.02–49.60	3.00	92.00
**6**	*y* = 0.1496*x* + 0.1560	0.9952	0.19–49.60	48.00	140.00
**7**	*y* = 0.0012 *x* + 0.0013	0.9938	0.78–49.60	193.00	640.00
**8**	*y* = 0.1692*x* + 0.2124	0.9920	0.05–49.60	12.00	48.00
**9**	*y* = 0.0042*x* + 0.0009	0.9985	0.19–49.60	97.00	260.00
**10**	*y* = 0.0325*x* − 0.0110	0.9952	0.10–49.60	12.00	34.00
**11**	*y* = 0.0661 *x* + 0.0919	0.9955	0.05–49.60	24.00	48.00
**12**	*y* = 0.0223*x* + 0.0342	0.9935	0.39–49.60	32.00	96.00
**13**	*y* = 0.0144*x* − 0.0040	0.9967	0.10–49.60	48.00	16.00
**14**	*y* = 0.1207*x* + 0.0956	0.9938	0.05–49.60	12.00	34.00
**15**	*y* = 0.0317*x* − 0.0026	0.9989	0.02–49.60	48.00	14.00
**17**	*y* = 0.3381*x* + 0.2201	0.9973	0.02–49.60	3.00	6.00
**18**	*y* = 0.3737*x* + 0.1974	0.9954	0.02–49.60	12.00	36.00
**19**	*y* = 0.6961*x* + 0.4415	0.9954	0.02–49.60	3.00	10.00
**20**	*y* = 0.6961*x* + 0.4415	0.9954	0.02–49.60	3.00	10.00
**21**	*y* = 0.3787*x* + 0.4894	0.9925	0.19–49.60	24.00	72.00
**22**	*y* = 0.4953*x* − 0.2220	0.9972	0.05–49.60	2.00	5.00

Peaks **1**–**22** were the same as Table 1. Peak **16** was the internal standard compound.

## Supplementary Materials

Supplementary Materials are available online.
