# Supplementary material for: A New UPLC-MS/MS Method for the Characterization and Discrimination of Polysaccharides from Genus Ephedra Based on Enzymatic Digestions
Source: Molecules. 2017 Nov 17;22(11):1992. doi: 10.3390/molecules22111992 (PMC6150222; doi:10.3390/molecules22111992)
Supplement: Supplementary file 1 [file molecules-22-01992-s001.pdf]

Appendix A. Supplementary data

A new UPLC-MS/MS method for characterization and discrimination of polysaccharides from genus *Ephedra* based on enzymatic digestions

Yong-Gang Xia, Tian-Long Wang, Li-Ming Sun, Jun Liang, Bing-You Yang and Hai-Xue Kuang*

Key Laboratory of Chinese Materia Medica (Heilongjiang University of Chinese Medicine), Ministry of Education, Harbin, 150040, P.R. China
* Correspondence: yonggangxia@163.com; Tel.: +86 45182767188

Academic Editor: name
Received: date; Accepted: date; Published: date

Supplementary Figure and Table Caption

Fig. S1. MRM ion chromatograms of 22 reference standards and blank.

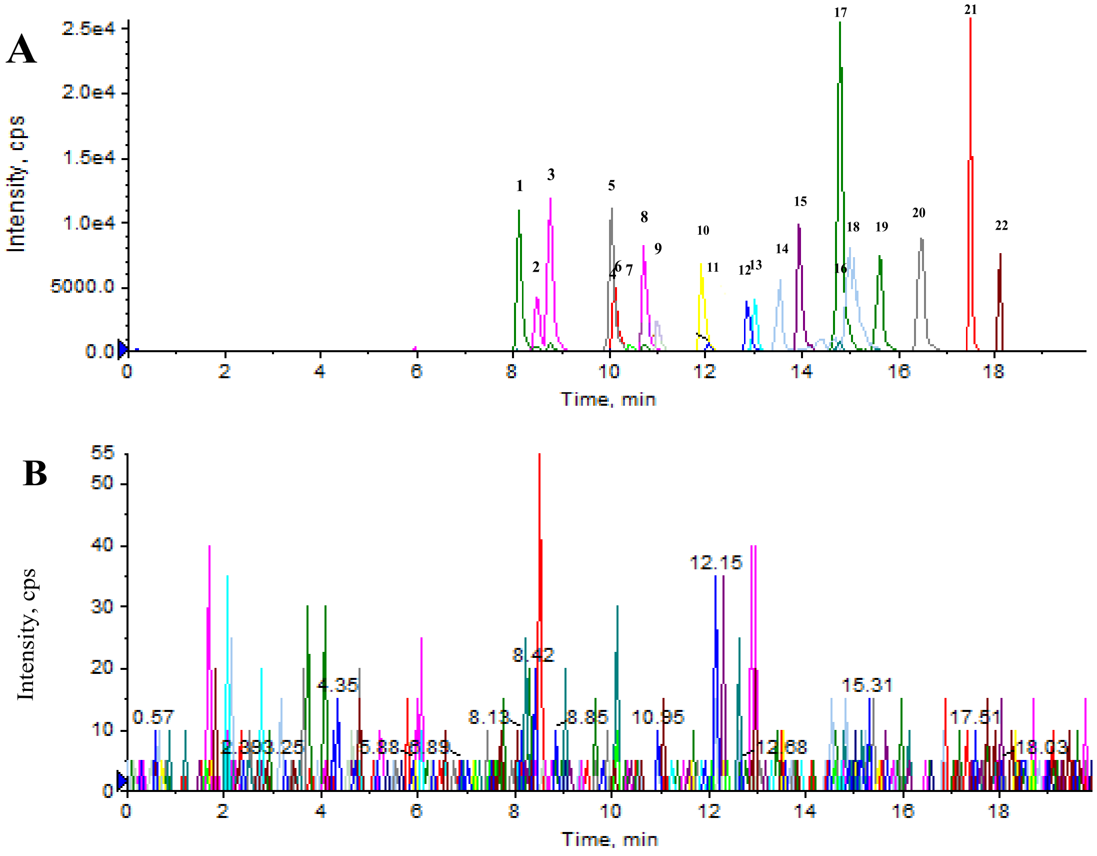

Table S1. Experimental results of saccharides liberated through specific enzymatic digestion of *Ephedra* herb.

Table S1

Samples	Batches	**mol%**									
		**Rib**	**Ara**	**Man**	**Glc**	**Gal**	**GlcUA**	**GalUA**	**Lactose**	**Maltotriose**	**Maltotetraose**
***endo*-1,4-*β*-Xylanase hydrolysis**											
E. sinica (root)	1	0.11	40.57	0.33	21.82	0.44	-	-	8.96	18.86	8.91
	2	0.09	48.83	0.38	15.50	0.43	-	-	12.04	15.86	6.86
	3	0.15	50.85	0.33	20.37	0.29	-	-	7.43	12.86	7.72
	4	0.17	51.38	0.36	17.19	0.39	-	-	8.99	12.18	9.34
	5	0.13	43.33	0.31	20.43	0.44	-	-	11.90	17.66	5.80
E. sinica (stem)	1	0.12	22.28	0.34	17.13	0.41	-	-	12.88	33.52	13.32
	2	0.10	16.85	0.46	20.00	0.35	-	-	16.01	31.84	14.38
	3	0.18	22.71	0.34	12.99	0.18	-	-	13.97	38.60	11.03
	4	0.14	21.52	0.41	21.02	0.33	-	-	14.99	33.68	7.91
	5	0.10	19.67	0.49	18.33	0.42	-	-	16.02	39.25	5.72

Samples	Batches	mol%									
		**Rib**	**Ara**	**Man**	**Glc**	**Gal**	**GlcUA**	**GalUA**	**Lactose**	**Maltotriose**	**Maltotetraose**
E. intermedia	1	0.08	23.97	0.55	16.08	0.61	-	-	12.18	35.86	10.68
	2	0.07	25.25	0.35	18.62	0.67	-	-	10.82	35.17	9.04
	3	0.16	23.80	0.36	19.85	0.46	-	-	10.34	35.28	9.75
	4	0.22	23.47	0.43	18.63	0.55	-	-	10.98	34.30	11.42
	5	0.17	22.31	0.30	17.00	0.67	-	-	13.45	35.24	10.86
E. equisetina	1	0.13	9.67	0.38	21.46	0.63	-	-	12.55	39.79	15.38
	2	0.08	11.77	0.37	21.56	0.37	-	-	12.91	37.43	15.51
	3	0.20	6.70	0.24	21.37	0.46	-	-	12.55	45.09	13.39
	4	0.17	8.67	0.30	21.09	0.53	-	-	11.97	42.94	14.43
	5	0.11	10.83	0.29	23.72	0.49	-	-	13.48	39.38	11.70
**Cellulose hydrolysis**											
E. sinica (root)	1	0.04	6.66	0.07	59.73	-	-	3.27	22.60	7.63	-
	2	0.02	5.05	0.04	70.06	-	-	2.84	17.64	4.35	-
	3	0.02	6.19	0.06	55.71	-	-	2.74	27.75	7.54	-
	4	0.02	5.77	0.05	66.34	-	-	3.02	25.49	5.24	-

Samples	Batches	mol%									
		**Rib**	**Ara**	**Man**	**Glc**	**Gal**	**GlcUA**	**GalUA**	**Lactose**	**Maltotriose**	**Maltotetraose**
	5	0.03	6.02	0.02	53.26	-	-	2.99	30.53	7.15	-
E. sinica (stem)	1	-	3.56	0.07	48.29	-	-	1.50	32.14	14.43	-
	2	-	2.80	0.05	55.18	-	-	1.05	28.41	12.50	-
	3	-	2.75	0.04	55.30	-	-	1.31	28.92	11.67	-
	4	-	2.94	0.08	53.45	-	-	1.43	30.58	11.52	-
	5	-	3.45	0.04	51.49	-	-	1.12	30.59	13.31	-
E. intermedia	1	-	4.16	0.03	45.37	-	-	1.27	33.84	15.34	-
	2	-	3.37	0.04	50.84	-	-	1.07	29.38	15.31	-
	3	-	3.82	0.03	45.53	-	-	1.21	32.07	17.34	-
	4	-	4.00	0.03	49.68	-	-	1.13	30.94	14.22	-
	5	-	3.67	0.03	51.35	-	-	1.09	31.48	12.38	-
E. equisetina	1	-	1.04	0.03	60.03	-	-	0.76	29.66	8.48	-
	2	-	1.08	0.03	56.34	-	-	1.22	31.25	10.08	-
	3	-	0.56	0.04	62.68	-	-	0.74	27.83	8.16	-
	4	-	0.89	0.04	61.19	-	-	0.95	30.02	6.91	-

Samples	Batches	mol%									
		**Rib**	**Ara**	**Man**	**Glc**	**Gal**	**GlcUA**	**GalUA**	**Lactose**	**Maltotriose**	**Maltotetraose**
	5	-	1.21	0.03	58.85	-	-	0.88	29.57	9.46	-
**α-Amylase hydrolysis**											
E. sinica (root)	1	-	1.13	0.06	22.53	0.11	-	-	56.69	19.48	-
	2	-	1.05	0.05	21.45	0.12	-	-	58.56	18.77	-
	3	-	0.85	0.05	21.48	0.08	-	-	65.47	12.07	-
	4	-	1.04	0.03	19.48	0.17	-	-	61.35	17.93	-
	5	-	1.08	0.04	18.77	0.13	-	-	60.03	19.95	-
E. sinica (stem)	1	-	0.43	0.05	19.92	0.09	-	-	57.70	21.81	-
	2	-	0.49	0.04	19.40	0.09	-	-	56.88	23.09	-
	3	-	0.38	0.07	18.14	0.09	-	-	60.10	21.22	-
	4	-	0.42	0.07	18.86	0.10	-	-	61.44	19.11	-
	5	-	0.38	0.05	17.35	0.09	-	-	58.39	23.74	-
E. intermedia	1	-	0.55	0.05	16.74	0.08	-	-	61.15	21.43	-
	2	-	0.58	0.03	16.36	0.09	-	-	60.92	22.01	-
	3	-	0.62	0.05	16.65	0.08	-	-	62.10	20.50	-

Samples	Batches	mol%									
		**Rib**	**Ara**	**Man**	**Glc**	**Gal**	**GlcUA**	**GalUA**	**Lactose**	**Maltotriose**	**Maltotetraose**
	4	-	0.60	0.04	16.33	0.08	-	-	61.88	21.07	-
	5	-	0.47	0.03	17.04	0.07	-	-	59.83	22.56	-
E. equisetina	1	-	0.39	0.05	19.45	0.05	-	-	62.77	17.28	-
	2	-	0.47	0.05	18.25	0.04	-	-	63.33	17.87	-
	3	-	0.45	0.05	18.67	0.08	-	-	63.47	17.27	-
	4	-	0.39	0.06	19.03	0.06	-	-	62.49	17.97	-
	5	-	0.40	0.04	18.28	0.07	-	-	60.35	20.86	-
***β*-(1→3)-D-Glucanase hydrolysis**											
E. sinica (root)	1	-	7.65	-	92.35	-	-	-	-	-	-
	2	-	4.31	-	95.69	-	-	-	-	-	-
	3	-	7.14	-	92.86	-	-	-	-	-	-
	4	-	6.67	-	93.33	-	-	-	-	-	-
	5	-	5.98	-	94.02	-	-	-	-	-	-
E. sinica (stem)	1	-	1.26	-	98.74	-	-	-	-	-	-
	2	-	1.58	-	98.42	-	-	-	-	-	-

Samples	Batches	mol%									
		**Rib**	**Ara**	**Man**	**Glc**	**Gal**	**GlcUA**	**GalUA**	**Lactose**	**Maltotriose**	**Maltotetraose**
	3	-	1.62	-	98.38	-	-	-	-	-	-
	4	-	1.44	-	98.56	-	-	-	-	-	-
	5	-	1.61	-	98.39	-	-	-	-	-	-
E. intermedia	1	-	17.73	-	82.27	-	-	-	-	-	-
	2	-	17.71	-	82.29	-	-	-	-	-	-
	3	-	19.58	-	80.42	-	-	-	-	-	-
	4	-	19.03	-	80.97	-	-	-	-	-	-
	5	-	17.66	-	82.34	-	-	-	-	-	-
E. equisetina	1	-	4.65	-	95.35	-	-	-	-	-	-
	2	-	4.77	-	95.23	-	-	-	-	-	-
	3	-	10.88	-	89.12	-	-	-	-	-	-
	4	-	6.87	-	93.13	-	-	-	-	-	-
	5	-	8.34	-	91.66	-	-	-	-	-	-
**Pectinase hydrolysis**											
E. sinica (root)	1	0.81	13.01	0.08	24.71	0.99	9.88	48.01	2.50	-	-

Samples	Batches	mol%									
		**Rib**	**Ara**	**Man**	**Glc**	**Gal**	**GlcUA**	**GalUA**	**Lactose**	**Maltotriose**	**Maltotetraose**
	2	0.75	10.92	0.08	26.24	1.07	9.42	48.80	2.71	-	-
	3	0.71	10.86	0.06	21.24	0.69	9.72	54.12	2.60	-	-
	4	0.66	12.24	0.08	23.37	1.01	10.13	50.48	2.03	-	-
	5	0.83	11.99	0.11	25.35	0.89	8.98	49.37	2.48	-	-
E. sinica (stem)	1	0.55	6.75	0.14	35.79	0.79	6.15	38.69	11.14	-	-
	2	0.55	6.49	0.14	35.82	0.86	7.99	38.49	9.56	-	-
	3	0.74	6.35	0.12	24.53	1.16	7.50	45.77	13.83	-	-
	4	0.61	6.26	0.13	34.23	0.93	6.89	42.48	8.47	-	-
	5	0.58	6.74	0.13	29.88	1.02	7.19	40.22	14.24	-	-
E. intermedia	1	0.73	8.66	0.14	34.00	1.20	7.17	41.60	6.50	-	-
	2	0.65	8.98	0.18	30.71	1.46	7.78	43.65	6.59	-	-
	3	0.68	9.15	0.13	34.53	1.03	8.86	37.17	8.46	-	-
	4	0.70	9.04	0.16	32.57	1.21	8.14	40.92	7.26	-	-
	5	0.63	8.73	0.13	31.79	1.09	7.79	39.08	10.76	-	-
E. equisetina	1	0.38	6.43	0.15	40.20	1.34	2.69	34.19	14.63	-	-

Samples	Batches	mol%									
		**Rib**	**Ara**	**Man**	**Glc**	**Gal**	**GlcUA**	**GalUA**	**Lactose**	**Maltotriose**	**Maltotetraose**
	2	0.39	4.97	0.16	43.38	1.24	2.44	32.44	14.98	-	-
	3	0.37	6.21	0.10	47.56	0.72	1.73	28.27	15.03	-	-
	4	0.36	5.53	0.11	44.30	1.13	2.31	30.04	16.22	-	-
	5	0.38	6.09	0.14	45.13	0.97	1.99	33.20	12.10	-	-
